# Intelligence

**Published:** 1826-10

**Authors:** 


### INTELLIGENCE.

### MONTHLY REPORT OF PREVALENT DISEASES.

Cases of diarrhœa, which were very frequent during the last three months, began to disappear in proportion as the heat of the weather abated, and few cases are now to be met with. Continued Fevers have, however, become so frequent as almost to constitute an epidemic, and in numerous instances the disease has assumed a severe form. The principal circumstance worthy of notice is the occurrence of gangrene or sloughing in a considerable proportion of cases. We have seen this in the upper part of the air-passages, in the posterior fauces and pharynx, and in the bowels; while in one instance a large carbuncle formed on the face. It is almost unnecessary to observe, that these occurrences have been extremely unfavourable: in most cases, indeed, their existence has only been discovered by post-mortem examination. The comparatively greater prevalence of continued fever at present than during the preceding part of the season, cannot be viewed without some anxiety, when taken in conjunction with the epidemic which has made its appearance in Dublin.

The only other change in the prevailing diseases, which is of sufficient importance to merit attention, is the increasing number of chronic rheumatic affections; the weather having been wet during a considerable portion of the present month, and the nights cold.

The frequency of Scarlatina and Measles is on the decrease; but we have seen a considerable number of cases of Small-pox.

*September 25th.*

---

*Royal College of Surgeons in London.—Bye-Law of the College, and Standing Orders of the Court of Examiners, relative to the age and professional education of Candidates for the Diploma.*

*Bye-Law.* Sect. xvi. § 1.—No person under twenty-two years of age shall be admitted a member of the College.

*Standing Orders.*—1. The only schools of anatomy and surgery recognised by the Court are, London, Dublin, Edinburgh, Glasgow, and Aberdeen.

2. Certificates of attendance upon the chirurgical practice of an hospital will not be received by the Court, unless such hospital be in one of the above recognised schools, and shall contain on an average 100 patients.

3. The Court will, however, receive, as testimonials of education, certificates of attendance on provincial hospitals, containing respectively 100 patients; provided a student shall have previously attended two courses of anatomical lectures, and two courses of dissections, in any of the recognised schools of anatomy. But the Court require that the term of attendance on such provincial hospitals shall be of twice the duration of that required at hospitals in any of the recognised schools of anatomy.

Candidates will, conformably to the above bye-law and standing orders, be required respectively to produce, prior to examination, certificates—

1. Of being twenty-two years of age.

2. Of having been engaged six years, at least, in the acquisition of professional knowledge.

3. Of having regularly attended three winter courses, at least, of lectures on anatomy and physiology, delivered at subsequent periods; and also one winter course, at least, of lectures on surgery.

4. Of having performed dissections during two or more subsequent winter courses.

5. And of having diligently attended, during the term of at least one year, the chirurgical practice of one of the following hospitals:—St. Bartholomew's, St. Thomas's, the Westminster, Guy's, St. George's, the London, and the Middlesex, in London; the Richmond, Steeven's, and the Meath, in Dublin; the Royal Infirmary, in Edinburgh; the Royal Infirmary, in Glasgow; or the Royal Infirmary, in Aberdeen: or of twice that term in any of the provincial hospitals, conformably to the above standing order, No. 3.

Such certificate must also express the dates of the commencement and of the termination of attendance on each course of lectures, and of dissections; and the periods of the commencement and of the termination of attendance on hospital practice.

The required certificates must be delivered at the College ten days, at least, prior to the day on which candidates shall, respectively, be desirous of admission to examination.

Candidates, under the following circumstances, and of the required age, are also admissible to examination:—Members of any of the legally-constituted Colleges of Surgeons in the United Kingdom; graduates in medicine of any of the Universities of the United Kingdom, who shall have performed two or more courses of dissection, as above specified, No. 4; and who shall have regularly attended the chirurgical practice of one of the hospitals, as above described, No. 5.

(By order,) EDMUND BELFOUR, *Sec.*

*Lincoln's Inn-fields; Sept. 8th,* 1826.

---

*Medical Benevolent Society.*—This Society was established in the year 1816, for the purpose of affording relief to such of its members "as are in distressed circumstances, from mental or bodily infirmity, or who, from other causes, shall be considered as requiring and deserving of assistance.

By a rule of the Society, no claim can be made on its fund till "after the expiration of ten years from the date of its establishment, when, but not sooner, relief may be granted."

The period, therefore, is now arrived when claims may be made; and the directors are anxious, as speedily and as effectually as possible, to exercise the discretionary power vested in them; but they have the mortification to find that the fund of the Society is very small,* whilst they have strong reasons for believing that the claims upon it will be numerous.

Instances of extreme necessity, to which medical practitioners of every rank have been reduced, are frequent and notorious. From the Reports of "The Society for the Benefit of the Widows and Orphans of Medical Men in London and its Vicinity," it appears that *one* in *four* of the families of its deceased members has received relief from that fund.

An attempt to explain the causes of the indigence of so many members of an useful and honourable profession, would be here irrelevant. It is, however, a melancholy fact, that such indigence does exist; which might probably have been prevented or mitigated by the establishment and liberal encouragement of institutions similar to this.

Although the Medical Benevolent Society has been honoured by a munificent and approving donation from his Majesty,—by the illustrious patronage of H. R. H. the Duke of Sussex,—by the benefactions of several noble and eminent persons,—and by the countenance of the most distinguished of the profession in the metropolis, still its fund is wholly inadequate to the attainment of its objects; and, although all medical men, throughout England and Wales, may be admitted amongst its members, yet the present number of them is in the proportion only of 250 to upwards of 12,000!

That a Society, constituted for a purpose so laudable, and upon principles so generally approved, and acted upon by other learned and scientific professions, should have been established for so many years, and yet have been so little encouraged, can only be accounted for on the supposition that its real objects and advantages have been either but partially known, or wholly misunderstood.

The directors of the Medical Benevolent Society, fully convinced of its utility, respectfully and earnestly, by this address, recommend it to the attention of the profession at large; and they trust that those who are raised above the contingencies of a medical life will, from motives of benevolence, afford it their patronage; and that the less affluent will, from motives of prudence, spare the trifling sum* required from their present incomes, in order to insure to themselves and to their families the means of assistance in the time of adversity.

* The capital is 2600*l.* sterling; the annual aggregate income is about 280*l.*

† The terms of admission are, one guinea on admission, and one guinea per annum in advance; or twenty guineas at one payment, constituting a life subscriber.

*Attendance on Lectures on Midwifery.*—We are sorry to find that the notice in our last Number on this subject was premature; the proposal to require certificates of attendance on midwifery lectures, of candidates for a licence to practise as Apothecaries, not having passed into a resolution. We can only say that those, a great part of whose business consists in the practice of midwifery, ought to give proof of their being properly qualified in this, as well as in the other departments of their profession. We would add, that the Apothecary's Company has thus lost an opportunity of showing their desire to improve still further the qualifications of those over whose education they have been appointed guardians, by a regulation salutary in itself, and which would have been approved of both by the profession and the community at large.

---

*Medical Education.*—We subjoin a list of the various Lectures about to commence, arranged according to subjects; from which it will be apparent, that, if the rising generation be deficient in any part of their education, it cannot be for want of a sufficient number of teachers.

Lectures will be delivered during the ensuing season, (or at least have been advertised,) on

*Anatomy, by*

Messrs. Abernethy and Stanley, St. Bartholomew's Hospital, commencing at half-past two p.m. Monday, October 2d.
Messrs. Bell and Mayo, Anatomical Theatre, Great Windmill-street, at two p.m. Monday, October 2d.—Mr. Hawkins, ditto, at one p.m. October 9th.
Mr. Bennett, Little Dean-street, Soho, at eleven a.m. Tuesday, October 3d.
Mr. Brookes, Anatomical Theatre, Blenheim-street, at twelve, October 2d.
Mr. Carpue, Dean-street, Soho, at two p.m. Monday, October 2d.
Mr. Dermott, Theatre, Windmill-street, Golden-square, at two p.m. ditto.
Mr. Grainger, Theatre of Anatomy and Medicine, Webb-street, Borough, ditto, ditto.
Messrs. Headington and Harkness, London Hospital, at two p.m.
Messrs. Green and South, St. Thomas's Hospital, at two p.m. Monday, Oct. 2d.
Mr. Kiernan, No. 38, Charterhouse-square, at eleven a.m. Monday, October 9th.
Messrs. Morgan and B. Cooper, Guy's Hospital, at two p.m.
Mr. Sleigh, Chapel-street, Grosvenor-square, at two p.m. Monday, October 2d.
Messrs. Tyrrell, Quain, and Coulson, Medical Theatre, No. 58, Aldersgate-street, at twelve, noon, ditto.

*On the Theory and Practice of Medicine, by*

Dr. Ager, Margaret-street, at nine a.m. Monday, October 2d.
Dr. Armstrong, *No.* 1, Anatomical and Medical Theatre, Webb-street, Borough, at four p.m. Monday, October 2d.
——————, *No.* 2, Theatre, Little Dean-street, Soho, at eight p.m. Tuesday, October 3d.
Dr. Ayre, Theatre of Anatomy and Medicine, Dean-street, Borough, four p.m. ditto.
Dr. Bright, Guy's Hospital, at half-past nine, Monday, October 2d.
Drs. Chambers and Macleod, Anatomical Theatre, Great Windmill-street, at nine a.m. Wednesday, October 4th.
Dr. Clutterbuck, assisted by Dr. Tweedie, Theatre, 58, Aldersgate-street, at ten a.m. ditto.
Dr. Copland, No. 17, Great Pulteney-street, at eight a.m. Monday, October 2d.
Dr. Gregory, No. 60, King-street, Golden-square, at nine a.m. October 4th.
Dr. Hue, St. Bartholomew's Hospital, at ten a.m. Tuesday, October 3d.
Dr. Milligan, at the Theatre, Chapel-street, Grosvenor-square, at eight a.m. Tuesday, October 3d.
Dr. Nuttall, at the Westminster Dispensary, Gerrard-street, Soho, at eight a.m. Monday, October 2d.
Dr. Ramadge, *No.* 1, at the Central Infirmary, Greville-street.
——————, *No.* 2, Theatre, Little Windmill-street, at nine a.m. October 2d.
Dr. Robinson, London Hospital, at nine, Tuesday, October 3d.
Drs. Shearman and Sigmond, 28, Villiers-street, Strand, Monday, October 2d.
Sir G. Tuthill, Cavendish-square, ditto ditto
Dr. Whiting, Surrey Dispensary, Union-street, Borough, at ten a.m. October 3d.

Drs. WILLIAMS and ELLIOTSON, St. Thomas's Hospital, at eleven A.M. October 3d.

*On Materia Medica, by*

Dr. ADDISON, Guy's Hospital, at seven P.M. Tuesday, October 3d.
Dr. BILLING, London Hospital, at half-past three, ditto.
Mr. JACKSON, No. 17, Webb-street, Maze Pond, at half-past six P.M. October 2d.

Dr. AGER,

Dr. BOOTT,

Dr. COPLAND,

Dr. GREGORY,

Dr. HUE and Mr. WHEELER,

Dr. MACLEOD,

Dr. MILLIGAN,

Dr. NUTTALL,

Dr. RAMADGE,

Dr. ROOTS,

Dr. SHEARMAN,

Dr. TWEEDIE,

These Lectures are connected with the corresponding courses on Medicine, and *for the most part* are delivered on the same days, and during the hour immediately preceding the Medical Lecture.

*On Surgery, by*

Mr. ABERNETHY, St. Bartholomew's Hospital, at eight P.M. Monday, October 2d.
Mr. BACOT, Theatre of Anatomy and Medicine, Dean-street, Borough, at seven P.M. Tuesday, October 10th.
Messrs. BELL and SHAW, Middlesex Hospital, at seven P.M. Tuesday, October 3d.
Mr. BRODIE, Anatomical Theatre, Great Windmill-street, at seven P.M. October 2d.
Mr. GREEN, St. Thomas's Hospital, at eight P.M. Monday, October 2d.
Mr. GUTHRIE, Westminster Infirmary for Diseases of the Eye, Warwick-street, at half-past six P.M. October 3d.
Messrs. HEADINGTON and LUKE, London Hospital, at eight P.M. on Monday, October 2d.
Messrs. KEY and MORGAN, Guy's Hospital, at eight P.M. Tuesday, October 3d.
Messrs. LAWRENCE and WARDROP, Medical Theatre, No. 58, Aldersgate-street, at seven P.M. ditto.

*On Midwifery, by*

Dr. BLUNDELL, Guy's Hospital, at a quarter before eight A.M.
Mr. CHOLMONDELEY, General Dispensary, Aldersgate-street, at a quarter-past eight P.M. Tuesday, October 3d.
Dr. CONQUEST and Mr. BIRCH, St. Bartholomew's Hospital, at half-past six P.M. Wednesday, October 4th
Dr. DAVID D. DAVIS, Theatre, 58, Aldersgate-street, at half-past five P.M. Monday, October 2d.
————————, 29, George-street, Hanover-square, at half-past ten A.M. Tuesday, October 3d.
Dr. HENRY DAVIES, Theatre, Dean-street, Borough, at nine A.M. ditto.
————————, Westminster Infirmary for Diseases of the Eye, Warwick-street, at a quarter-past ten A.M. Monday, October 2d.
Dr. GOLDING, Villiers-street, Strand, ditto.
Dr. GRANVILLE, Westminster Dispensary, Gerrard-street, Soho.
Dr. HOPKINS, Theatre, Webb-street, Borough, at four P.M. Tuesday, October 3d.
Mr. JACKSON, No. 17, Webb-street, Maze Pond, at half-past six P.M. ditto.
Mr. JEWEL, Middlesex Infirmary, Great Pulteney-street, Golden-square, at eight P.M. October 11th.
Dr. LEY, Middlesex Hospital, at nine A.M. Monday, October 9th.
Dr. LOCOCK, St. Thomas's Hospital, at eight A.M. Tuesday, October 3d.
Mr. MAXFIELD, Central Infirmary, Greville-street, Hatton Garden.
Dr. POWER, Theatre of Anatomy, 23, Chapel-street, Grosvenor-square, at nine A.M. Tuesday, October 3d.
Dr. RAMSBOTHAM and Dr. F. RAMSBOTHAM, London Hospital, at ten A.M. Wednesday, October 4th.
Mr. STONE, Anatomical Theatre, Great Windmill-street, at a quarter-past ten, Wednesday, October 4th.
Mr. WALLER, No. 111, Aldersgate-street, at a quarter-past eight P.M. Tuesday, October 10th.

*On Chemistry, by*

| | |
|---|---|
| Dr. Ager,<br>Dr. Clutterbuck,<br>Dr. Copland,<br>Dr. Hue and Mr. Wheeler,<br>Dr. Ramadge,<br>Sir G. Tuthill, | These gentlemen give courses of Chemistry connected with their Lectures on Medicine, and *generally* on the alternate days. |

Messrs. Allen and Aikin, and Dr. Bostock, Guy's Hospital, at half-past nine, Tuesday, October 3d.
Messrs. Brande and Faraday, at the Royal Institution, Albemarle-street, at nine a.m. Tuesday, October 10th.
Dr. Birkbeck and Mr. Pereira, General Dispensary, Aldersgate-street, at ten p.m. Tuesday, October 3d.
Mr. Burton, St. Thomas's Hospital, at eleven a.m. Monday, October 2d.
Mr. Cooper, No. 58, Aldersgate-street, ditto ditto.
Dr. Gordon, London Hospital, at ten a.m. Tuesday, October 3d.
Mr. Maxfield, Central Infirmary, Greville-street, Monday, October 2d.
Mr. Maugham, No. 1, Dean-street, Borough, at half-past six p.m. ditto
Mr. Phillips, Theatre, Webb-street, Borough, at a quarter before ten a.m. Tuesday, October 3d.
Mr. Wood, Anatomical Theatre, Great Windmill-street, at nine a.m. ditto.

*On Physiology, by*

Dr. Barry, *No.* 1, Theatre, Little Dean-street, Soho.
————, *No.* 2, Theatre, Webb-street, Borough.
Dr. Blundell, Guy's Hospital, at half-past six p.m. Monday, October 2d.
Dr. Roget, Theatre, 58, Aldersgate-street, at eleven a.m. Tuesday, October 3d.
Dr. Southwood Smith, Theatre, Webb-street, Borough, at half-past six p.m. Monday, October 2d.

*On Anatomy, Physiology, and Diseases of the Ear, by*

Mr. Curtis, Royal Dispensary, Dean-street, Soho, at seven p.m. October 2d.

*On Cutaneous Diseases, by*

Dr. Ashby Smith, No. 12, Bloomsbury-square, at nine, Thursday, October 19th.

*On the Structure and Diseases of the Teeth, by*

Mr. Thomas Bell, Guy's Hospital.
Mr. J. Snell, 11, Crawford-street, Montague-square, at seven p.m. October 6th.

*On Botany, by*

Dr. Bright, Guy's Hospital.
Dr. Lush, St. Thomas's Hospital, at half-past twelve, Thursday, October 5th.

*On Experimental Philosophy, by*

Messrs. Allen and Millington, Guy's Hospital, at half-past five p.m. Thursday, October 5th.

---

*New Bartholomew School.*—In answer to our Correspondent, who is desirous of obtaining further information about this School, we beg leave to refer him to the very lucid Prospectus to be found in our last Number. His doubts about the selection of the teachers, appear to us quite unreasonable. Is it not stated in the document alluded to,—so remarkable for its unostentatious development of the plan,—not only that they *were* selected, but "*selected solely on the ground of their fitness for the departments allotted to them?*"! and, with respect to some of them, that their talents "*are so well known as to render any account of the manner in which the duties will be performed, perfectly unnecessary*"!!! What more would our Correspondent have? Surely nothing can be in better taste: nothing more satisfactory, than such testimony coming from the parties themselves, who are evidently the best judges of their own pretensions, and indeed the very persons on whose impartiality the discerning part of the profession will be disposed to rely.

---

*Hunterian Museum.*—We inserted a letter on this subject in our last Number, and have received another signed by "a Physician." We have at all times a dislike to anonymous communications, as in most cases the editor of a Journal gets the credit (or discredit) of them himself. But on the present occasion we have an additional motive for declining to insert the paper in question,—namely, that the writer carries his censures much further than we can go along with him. He states, indeed, that his address is intended "neither to excite clamour nor to encourage prejudices:" to us, however it appears calculated to do both. We are not among those who are disposed to "laugh at the College," on the one hand, or "to truckle to the Fellows," on the other: in fact, we have avoided taking any part in medical politics, and in our last Number we alluded to an act of the *Censors* alone, whose officious interference with the College of Surgeons we believe to be condemned by none more heartily than by the members of their own body. On the subject which has called forth these observations, we repeat that explanation is required. Either it did rest with the Censors to have the Licentiates admitted to the Hunterian Museum, or it did not. If it did not depend upon them, let it be made so to appear, and no impartial person will blame them in this particular instance, whatever he may think of their taste and judgment in interfering in the business at all: if it did rest with them to admit or exclude the Licentiates, and they preferred the latter, then nothing that has been said of them is too severe, and nothing that *we* have said is severe enough.

---

After the Number had been completed, and when the last half-sheet was on the very eve of being thrown off, we received the following letter: as it is in allusion to the subject of the preceding paragraph, we have, though with some difficulty, made arrangements for its insertion.

Sir,—There is a letter in the last Number of your Journal, from "a Licentiate," which you think requires explanation on the part of the Censors of the College of Physicians.* It contains a complaint that the privilege of introducing visiters to the Hunterian Museum is restricted to the Fellows of the College of Physicians and the members of the College of Surgeons. I hardly think such explanation necessary; for surely every liberal and intelligent person would conclude that the regulation did not proceed from them.† However, I can have no objection to its being stated on my part, that it was one of the original conditions, comprised in a Treasury minute, when the Museum was presented to the College of Surgeons, which the trustees were merely appointed to see enforced. I am, &c.

*Margaret-street; Sept. 27th,* 1826. JOSEPH AGER.

---

*Literary Notice.*—In the press, Dr. N. Arnott's work on General and Medical Physics. It is a system of natural and experimental philosophy, with strictly scientific arrangement, but without any of the technicalities which render the study difficult to persons unacquainted with mathematics. It is intended to comprise all in natural philosophy that a man of liberal education requires to know, or that has any bearing on the medical art. Many questions of physiology, pathology, and practice, are fully entered into; there is a detailed account of late improvements both in theory and practice, and there are some new suggestions.

* It was not the *letter* of the Licentiate, but the *conduct* of the Censors, which we said required explanation.

† We never said or supposed that the regulation proceeded from the *Fellows of the College of Physicians* or the *Members of the College of Surgeons;* but we inquired whether the exclusion of the Licentiates, on a recent occasion, did or did not originate with the *Censors,* as trustees *ex officio.*

### METEOROLOGICAL JOURNAL,

*From August 20th, to September 20th, 1826.*

By Messrs. Harris and Co. Mathematical Instrument Makers, 50, High Holborn.

| Aug. | Moon. | Rain gauge. | Thermom. | | | Barometer. | | De Luc's Hygrom. | | Winds. | | Atmospheric Variations. | | |
|---|---|---|---|---|---|---|---|---|---|---|---|---|---|---|
| | | | 9 A.M. | MAX. | MIN. | 9 A.M. | 10P.M. | 9 A M. | 10P.M. | 9 A.M. | 10PM. | 9 *a.m.* | 2 *p.m.* | 10 *p.m.* |
| 20 | | | 70 | 85 | 61 | 29.89 | 29.77 | 72 | 50 | E | SW | Fine | Fine | Fine |
| 21 | | | 66 | 73 | 60 | 29.79 | 29.77 | 68 | 75 | N | E | Cloudy | —— | Cloudy |
| 22 | | | 69 | 74 | 62 | 29.74 | 29.73 | 65 | 73 | WSW | SW | Fair | —— | Fair |
| 23 | | | 70 | 73 | 63 | 29.65 | 29.51 | 71 | 80 | SSW | S | Cloudy | Cloudy | Sm. Ra. |
| 24 | | | 68 | 75 | 64 | 29.57 | 29.62 | 67 | 73 | SW | SW | Fair | Fair | Fair |
| 25 | ☾ | .16 | 71 | 80 | 62 | 29.60 | 29.47 | 71 | 70 | SSW | SSW | —— | —— | Th.& L. |
| 26 | | | 69 | 73 | 56 | 29.73 | 29.72 | 67 | 65 | SW | SW | —— | —— | Fine |
| 27 | | | 66 | 71 | 57 | 29.78 | 29.86 | 68 | 68 | WNW | SW | —— | —— | —— |
| 28 | | | 69 | 76 | 65 | 29.84 | 29.79 | 67 | 79 | SSE | SSW | —— | —— | —— |
| 29 | | | 70 | 76 | 63 | 29.71 | 29.65 | 75 | 75 | SW | SE | Cloudy | —— | —— |
| 30 | | | 75 | 79 | 63 | 29.57 | 29.58 | 62 | 75 | S var. | SW | Fair | —— | Cloudy |
| 31 | | .10 | 67 | 73 | 59 | 29.65 | 29.65 | 67 | 74 | W | SSW | Fine | —— | Rain |
| Sept. | | | | | | | | | | | | | | |
| 1 | | | 66 | 70 | 58 | 29.63 | 29.62 | 72 | 78 | E | NE | Fair | —— | —— |
| 2 | ● | .30 | 59 | 65 | 61 | 29.55 | 29.57 | 88 | 92 | NEva. | N | Rain | —— | Cl.&Ra. |
| 3 | | | 64 | 67 | 59 | 29.62 | 29.75 | 80 | 79 | ESE | SE | Fair | Clo. Ra. | Cloudy |
| 4 | | | 68 | 73 | 58 | 29.75 | 29.76 | 73 | 82 | SSW | WSW | —— | Rain | —— |
| 5 | | | 65 | 66 | 54 | 29.76 | 29.67 | 70 | 69 | NW | WNW | Cloudy | Show'ry | —— |
| 6 | | | 57 | 65 | 52 | 29.33 | 29.17 | 91 | 73 | E | WSW | Rain | Rain | —— |
| 7 | | 100 | 54 | 56 | 50 | 29.12 | 29 61 | 87 | 75 | W | WNW | —— | —— | —— |
| 8 | | | 55 | 67 | 52 | 29.57 | 29.65 | 88 | 83 | SW | W | —— | —— | Fair |
| 9 | ☽ | .49 | 53 | 62 | 50 | 29.73 | 29.82 | 87 | 75 | E | WNW | —— | Show'ry | —— |
| 10 | | | 59 | 65 | 50 | 29.96 | 30.07 | 74 | 68 | W | W | Fair | Fine | Cloudy |
| 11 | | | 55 | 64 | 49 | 30.12 | 30.18 | 76 | 72 | W | W | —— | —— | Fair |
| 12 | | | 60 | 66 | 48 | 30.15 | 30.09 | 71 | 71 | W | W | Fine | —— | Fine |
| 13 | | | 54 | 66 | 52 | 30.02 | 29.91 | 76 | 72 | SW | W | —— | —— | —— |
| 14 | | | 60 | 69 | 47 | 29.85 | 30.01 | 76 | 76 | SW | NE | —— | Rain | Cloudy |
| 15 | | | 55 | 63 | 48 | 30.14 | 30.24 | 72 | 70 | NE | E | Fair | Fine | Fine |
| 16 | ○ | | 52 | 64 | 51 | 30.21 | 30.05 | 76 | 76 | E | ESE | —— | —— | —— |
| 17 | | | 63 | 71 | 57 | 29.92 | 29.87 | 74 | 86 | ESE | ESE | Fine | —— | —— |
| 18 | | .50 | 62 | 67 | 61 | 29.77 | 29.78 | 93 | 97 | ENE | ENE | Rain | Rain | Fair |
| 19 | | | 63 | 68 | 59 | 29.81 | 29.84 | 88 | 88 | ESE | E | Fair | Fine | Fine |

The quantity of Rain fallen in the month of August, was 2 inches and 27.100ths.

